# Loss of CD147 results in impaired epithelial cell differentiation and malformation of the meibomian gland

**DOI:** 10.1038/cddis.2015.98

**Published:** 2015-04-16

**Authors:** J Mauris, J Dieckow, S Schob, B Pulli, M P Hatton, S Jeong, A Bauskar, E Gabison, R Nowak, P Argüeso

**Affiliations:** 1 Schepens Eye Research Institute and Massachusetts Eye and Ear, Harvard Medical School, Boston, MA, USA; 2 Center for Systems Biology, Massachusetts General Hospital, Harvard Medical School, Boston, MA, USA; 3 Ophthalmic Consultants of Boston, Boston, MA, USA; 4 Institute for Genetic Medicine and Department of Ophthalmology, Keck School of Medicine, University of Southern California, Los Angeles, CA, USA; 5 Institute for Genetic Medicine and Graduate Program in Medical Biology, Keck School of Medicine, University of Southern California, Los Angeles, CA, USA; 6 Cornea and External Diseases, Fondation A. De Rothschild, Hôpital Bichat, APHP, Université Paris VII Diderot, Paris, France; 7 Department of Animal Sciences, University of Illinois, Urbana, IL, USA

## Abstract

Meibomian gland dysfunction is a leading cause of ocular surface disease. However, little is known about the regulatory processes that control the development and maintenance of this sebaceous gland. Here, we identify a novel function for CD147, a transmembrane protein that promotes tissue remodeling through induction of matrix metalloproteinases, in regulating meibocyte differentiation and activity. We found that CD147 localized along basal cells and within discrete membrane domains of differentiated meibocytes in glandular acini containing gelatinolytic activity. Induction of meibocyte differentiation *in vitro* promoted CD147 clustering and MMP9 secretion, whereas RNAi-mediated abrogation of CD147 impaired MMP9 secretion, concomitant with a reduction in the number of proliferative cells and cytoplasmic lipids. Meibomian glands of CD147 knockout mice had a lower number of acini in both the superior and inferior tarsal plates of the eyelids, and were characterized by loss of lipid-filled meibocytes compared with control mice. Together, our data provide evidence showing that gelatinolytic activity in meibocytes is dependent on CD147, and supports a role for CD147 in maintaining the normal development and function of the meibomian gland.

Sebacious glands are holocrine lobulated structures found over most surfaces of the human body.^1^ Traditionally thought to synthesize and secrete lipids to promote barrier function of the skin against environmental insult, emerging research has provided evidence of their involvement in additional functions such as regulation of immunological functions and inflammatory processes.^2^ Meibomian glands constitute a specialized form of sebaceous gland located inside the tarsal plate of the eyelids, where they promote stability of the tear film and prevent ocular surface dryness.^3^ Impairment in sebaceous gland function has been associated with a variety of diseases, which in the eye include meibomian gland dysfunction, a disease affecting millions of people worldwide.^3, 4, 5^ Complete absence of meibomian glands has been reported in patients with ectodermal dysplasia, ectrodactyly, and clefting syndrome and anhidrotic ectodermal dysplasia, rare congenital conditions characterized by abnormal ectoderm development.^6^

The structure and secretory composition of the meibomian glands has been extensively studied, both under physiological and pathological conditions. By comparison, little is known about the molecular mechanisms that regulate meibomian gland development and maintenance.^7^ The meibomian gland, as any other holocrine gland, undergoes constant self-renewal. Critical to maintain tissue architecture is the extracellular matrix, a major component of the cellular microenvironment.^8^ Alteration of the extracellular matrix occurs when radical remodeling is needed, such as during organ development and tissue homeostasis, and is associated with changes in cell proliferation and differentiation. The most significant enzymes involved in the degradation and remodeling of the extracellular matrix in tissues are zinc-dependent matrix metalloproteinases (MMPs) that include gelatinase B or MMP9. Both three-dimensional culture models and genetic manipulations of mice carrying gain- or loss-of-function alleles of MMPs have shown a critical role for MMPs in gland development and branching morphogenesis.^9, 10^ At the ocular surface, the homeodomain transcription factor *Barx2* has been shown to control gland morphogenesis by inducing the expression of MMPs.^11^ However, the exact mechanism underlying the expression of MMPs in ocular glands is not yet clear.

The production and activation of MMPs are tightly regulated by complex mechanisms that include the extracellular matrix metalloproteinase inducer CD147 (also known as basigin in the mouse), a glycosylated type I transmembrane protein known to induce cell migration and invasion.^12, 13^ CD147 activity was initially discovered in 1982 in a series of studies showing that co-culture of tumor cells and fibroblasts lead to stimulation of MMP1 production.^14, 15^ Since then, additional evidence has demonstrated that homophilic CD147 oligomerization induces MMP production, both through heterotypic and homotypic cell interactions.^13^ The molecular weight of CD147 is highly variable (31–65 kDa) because of heterogeneous *N*-glycosylation, with the most highly glycosylated forms functioning to induce MMP production.^16^ Importantly, CD147 has been proposed as a modulator of MMP expression during development and cell differentiation.^17^ In support of this hypothesis are findings showing strong induction of CD147 in basal and suprabasal cells during skin organogenesis.^18^

Here, we analyze the distribution of CD147 in the human meibomian gland and its contribution to MMP production. We find that CD147 is expressed in basal epithelial cells and within discrete membrane domains of differentiated meibocytes, and that it regulates MMP9 biosynthesis during meibocyte differentiation. Moreover, we show that CD147 modulates meibocyte proliferation and lipid biosynthesis. Finally, we use CD147 knockout mice as a model system to examine the relevance of these findings to meibomian gland formation and activity *in vivo*.

## Results

### CD147 and gelatinolytic activity localize to acinar structures in human meibomian glands

**Figure 1:**
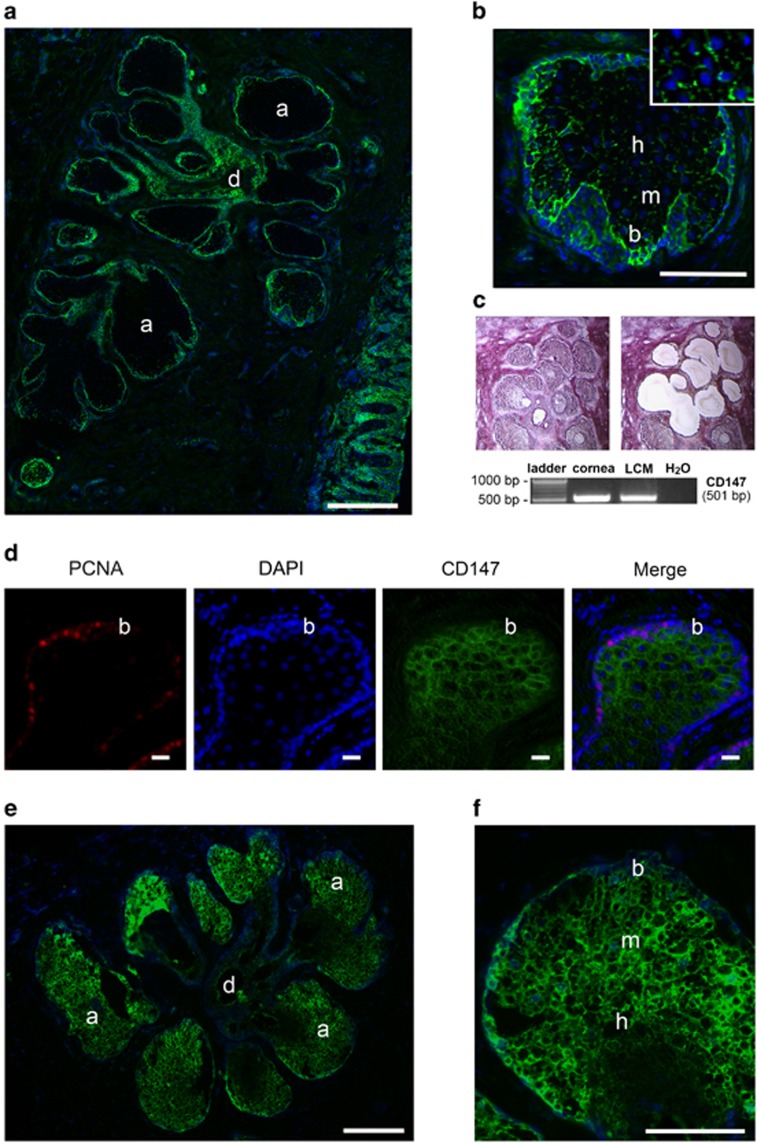
CD147 and gelatinolytic activity localize to acinar structures in human meibomian glands. (**a**) Immunofluorescence microscopy of a human eyelid showing CD147 localization (green) to basal cells at the peripheral margin of secretory acini and to the multilayered squamous epithelium of the ductule. Nuclei were counterstained using DAPI (blue). (**b**) Within the acinus, CD147 staining decreased towards differentiated mature and hypermature meibocytes. This central region was characterized by patchy staining of CD147 along the cell membrane (inset). (**c**) Laser capture microdissection (LCM) and RT-PCR analysis demonstrating CD147 mRNA in acinar structures of meibomian gland tissue. Representative micrographs are shown before (*left*) and after (*right*) microdissection. Cultures of human corneal epithelial cells were used as positive control. (**d**) Immunofluorescence microscopy of a human eyelid showing PCNA (red) localization to basal acinar cells. CD147 was labeled in green. Nuclei were counterstained using DAPI (blue). (**e**) *In situ* gelatin zymography showing localization of gelatinolytic activity in secretory acinar cells of the meibomian gland. (**f**) Within the acinus, activity was detected in basal and mature meibocytes, and decreased towards the disintegrating zone. Acinus (a), basal cell (b), ductile (d), epidermis (e), mature cell (m), hypermature cell (h). Scale bars, 300 *μ*m (**a**), 10 *μ*m (**d**), 200 *μ*m (**e**), 100 *μ*m (**b** and **f**)

To determine whether CD147 modulates the expression of MMPs in meibomian glands, we first evaluated the distribution of CD147 in upper eyelids of human donors by immunofluorescence microscopy. Meibomian glands are composed of clusters of secretory acini that are arranged circularly around a long central duct and connected to it by short ductules.^19^ By immunofluorescence, CD147 localized to basal cells at the peripheral margin of secretory acini and to the multilayered squamous epithelium of the ductule (Figure 1a). While basal, proliferating meibocytes stained distinctly for CD147, the signal decreased towards differentiated mature and hypermature meibocytes (Figure 1b). This central region was characterized by patchy or discontinuous staining of CD147 along the cell membrane (Figure 1b, inset). The presence of CD147 mRNA in acinar structures was demonstrated by laser capture microdissection of meibomian gland tissue using RT-PCR, and further confirmed by cDNA sequencing (Figure 1c). The presence of proliferating basal acinar cells was demonstrated by staining for proliferating cell nuclear antigen (PCNA), a marker of growing cells entering the early S phase of the cell cycle (Figure 1d).^20^

In order to evaluate the role of CD147 in promoting MMPs in human meibocytes, we then examined the distribution of gelatinolytic activity throughout the meibomian gland. *In situ* gelatin zymography revealed localization of gelatinolytic activity primarily in secretory acinar structures within the meibomian gland (Figure 1e). The activity was detected in basal and intermediate cell layers, representing proliferating and mature meibocytes, respectively, and decreased towards the disintegrating zone, located at the transition of the acinus to the ductule (Figure 1f).

### CD147 promotes MMP9 biosynthesis during meibocyte differentiation

**Figure 2:**
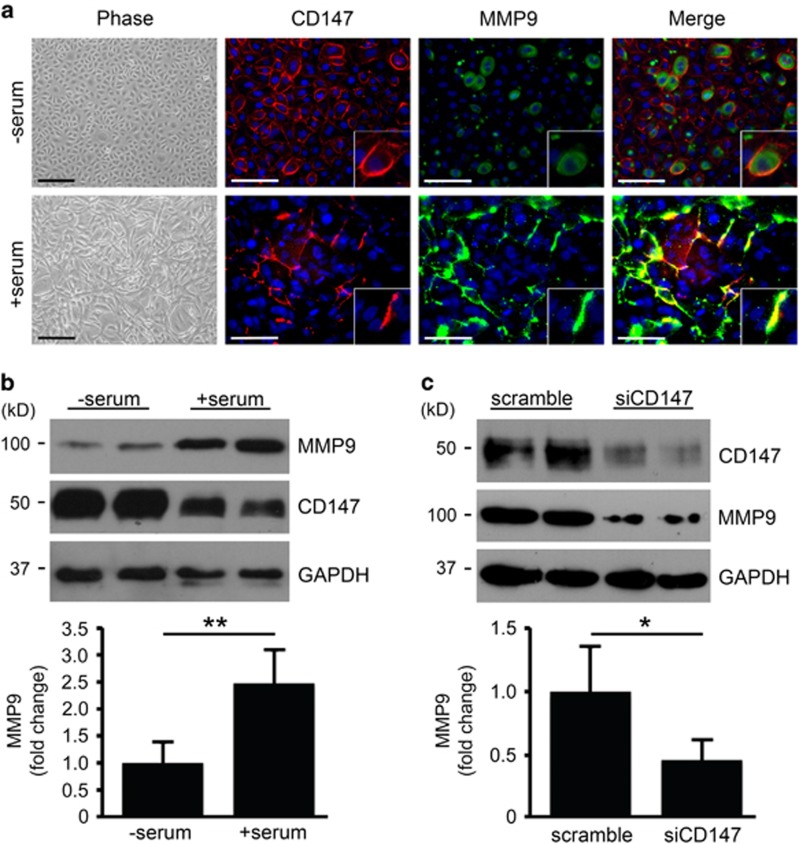
CD147 promotes MMP9 biosynthesis during meibocyte differentiation. (**a**) By phase-contrast microscopy, cultured meibocytes in serum-free media proliferated and had cobblestone-like morphology, whereas addition of serum induced differentiation and stratification. When grown in serum-free media, CD147 had a continuous pericellular distribution (red) while MMP9 (green) was primarily intracellular. Addition of serum promoted a discontinuous pattern of CD147 staining along the cell membrane that colocalized with areas that were positive for MMP9 (inset). Nuclei were counterstained using DAPI (blue). Scale bars, 200 *μ*m (phase), 100 *μ*m (immunofluorescence). (**b**) Immunoblot showing increased levels of MMP9 in whole cell lysates after addition of serum to cell cultures. Levels of CD147, on the other hand, decreased upon serum-induced differentiation. Data shown are representative of two independent experiments and the experiments were performed at least in duplicate (mean±S.D.; **P<0.01, Student's *t*-test). (**c**) Biosynthesis of MMP9 decreased in differentiated meibocytes following abrogation of CD147 using siRNA. Data shown are representative of two independent experiments and the experiments were performed at least in duplicate (mean±S.D.; *P<0.05, Student's *t* test)

To test a direct link between CD147 and MMP9 biosynthesis during meibocyte differentiation, we took advantage of an experimental cell culture model system using hTERT-immortalized human meibomian gland epithelial cells.^21^ In this system, confluent cells in serum-free media proliferate and have cobblestone-like morphology, whereas addition of serum induces differentiation and stratification (Figure 2a).^21^ Immunolocalization studies in cells grown in serum-free media revealed continuous pericellular distribution of CD147 throughout the cell culture (Figure 2a). Under these conditions, MMP9 had preferentially an intracellular distribution. Interestingly, induction of differentiation and stratification in the cell culture was characterized by patchy or discontinuous staining of CD147 along the cell membrane, reminiscent of the staining observed in mature meibocytes within acinar structures *in vivo*. Moreover, addition of serum to the cell culture resulted in CD147 colocalization with areas that were positive for MMP9 staining (Figure 2a), suggesting a link between the two proteins during meibocyte differentiation.

To determine whether CD147 was responsible for MMP9 biosynthesis and secretion in human meibocytes *in vitro*, we silenced CD147 using small interfering RNA (siRNA). As evidenced by immunoblot, addition of serum to cell cultures increased the levels of MMP9 in whole cell lysates (Figure 2b). The levels of CD147, on the other hand, decreased upon serum-induced differentiation, concomitant to the observation in Figure 2a showing discontinuous staining of CD147 along the cell membrane. Importantly, MMP9 biosynthesis was impaired in differentiated cells following treatment with siRNA targeting CD147 compared with scramble control (Figure 2c), suggesting an important role for CD147 expression and clustering along the cell membrane of differentiated meibocytes in promoting MMP9 biosynthesis.

### CD147 regulates meibocyte proliferation and lipid biosynthesis

**Figure 3:**
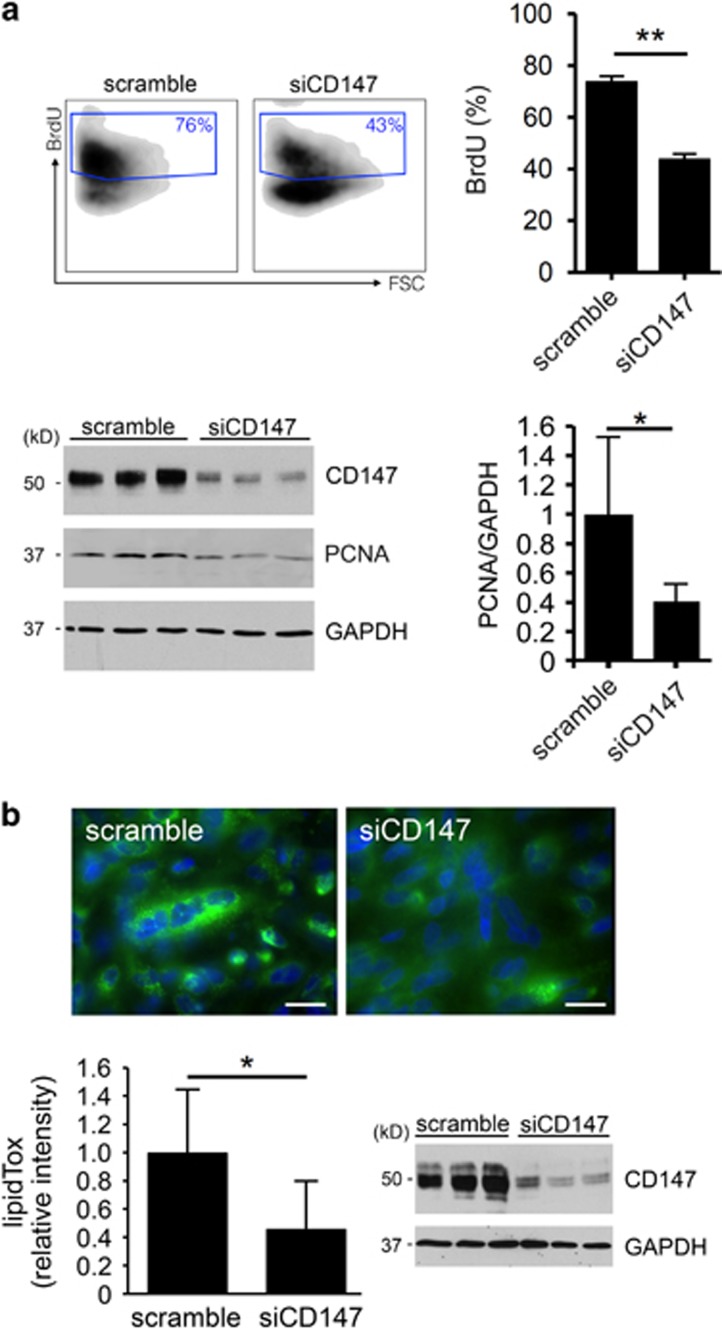
CD147 regulates meibocyte proliferation and lipid biosynthesis. (**a**) BrdU incorporation was used to assess cell cycle progression. As shown by flow cytometry, treatment of human meibocytes grown in serum-free media with siRNA targeting CD147 led to a 30% decrease in the incorporation of BrdU compared with scramble control. Each of the samples was analyzed in sixtuplicate (mean±S.D.; **P<0.01, Mann Whitney test). Immunoblot experiments demonstrate decreased biosynthesis of CD147 and a 60% decrease in PCNA in human meibocytes grown in serum-free media following abrogation of CD147 using siRNA. Experiments were performed in triplicate (mean±S.D.; *P<0.05, Student's *t*-test). FSC, forward scatter. (**b**) Abrogation of CD147 using siRNA impaired intracellular accumulation of neutral lipids in differentiated meibocytes. Representative images for each condition are shown in the upper panel. Experiments were performed in triplicate (mean±S.D.; *P<0.05, Student's *t*-test). Immunoblot experiments demonstrate decreased biosynthesis of CD147 in differentiated meibocytes following abrogation of CD147 using siRNA. Scale bars, 20 *μ*m

In addition to its role in mediating MMP biosynthesis, data published so far suggest that CD147 also has additional functions that include promotion of cellular proliferation.^22, 23^ Therefore, we examined the hypothesis that CD147 interferes with the cell cycle progression of human meibocytes. For these experiments, we used bromodeoxyuridine (BrdU), a pyrimidine analog that incorporates into newly synthesized DNA of actively proliferating cells. As shown in Figure 3a, incorporation of BrdU decreased after treatment of human meibocytes with siRNA targeting CD147, supporting the concept that CD147 positively regulates cell proliferation in human meibocytes. Consistent with reduced BrdU incorporation, we also observed a decrease in PCNA in protein lysates of cells treated with CD147 siRNA. Further, the intracellular accumulation of neutral lipids was impaired following abrogation of CD147 (Figure 3b), suggesting that CD147 expression also contributes to promote meibocyte differentiation.

### Bsg^−/−^ mice show morphological and functional alterations in the meibomian glands

**Figure 4:**
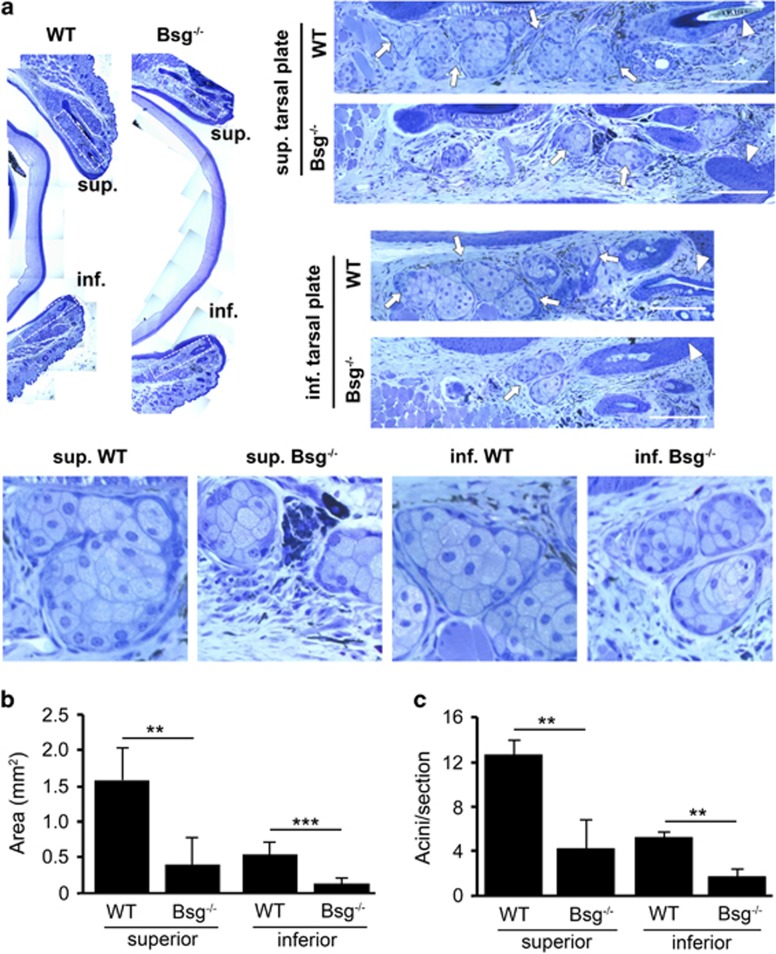
Meibomian glands in Bsg^−/−^ mice show altered morphology. (**a**) Histological analysis of toluidine blue-stained sections of tarsal plates (dashed rectangles) in the upper and lower eyelids. WT control mice (WT; *n*=4) contained clusters of secretory acini (arrows) throughout the length of the superior and inferior tarsal plates. By comparison, secretory acini in meibomian glands of Bsg^−/−^ mice (*n*=4) were poorly developed and appeared smaller in size. The arrowhead indicates the terminal position of the central meibomian duct at the eyelid margin. The lower panel contains magnified images of secretory acini areas. Scale bars, 50 *μ*m. (**b** and **c**) Quantification of the total area and number of secretory acini per section in the superior and inferior tarsal plates of WT and Bsg^−/−^ mice. A total of 25–50 sections were analyzed per eyelid sample (mean±S.D.; **P<0.01, ***P<0.001, Student's *t*-test)

**Figure 5:**
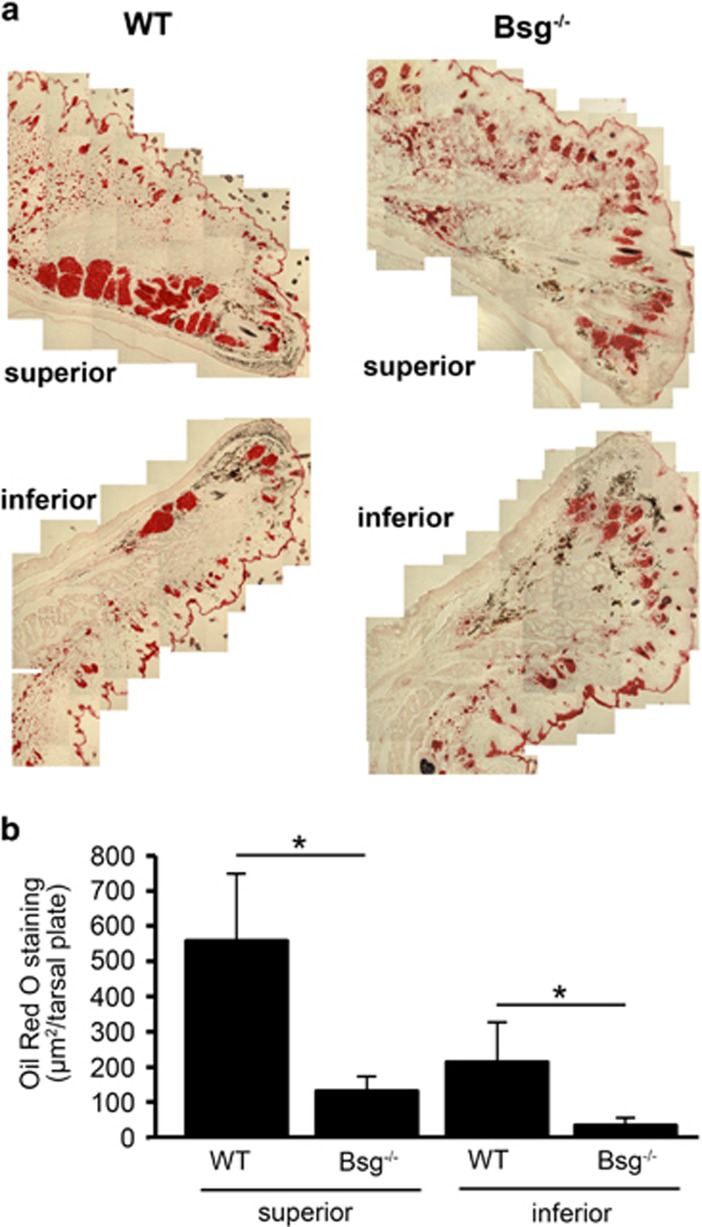
Bsg^−/−^ mice show functional alterations in the meibomian glands. (**a**) Oil Red O staining shows abundant lipid deposition in the superior and inferior tarsal plates of the meibomian glands in WT control mice (WT) as compared with Bsg^−/−^ mice. (**b**) Quantification of areas of Oil Red O staining per tarsal plate in WT (*n*=3) and Bsg^−/−^ (*n*=3) mice using ImageJ. A total of 16–32 sections were analyzed per eyelid sample (mean±S.D.; *P<0.05, Student's *t*-test)

Homozygous knockout mice (Bsg^−/−^) and age-matched littermate controls (WT) were examined to determine the relevance of CD147 expression to meibomian gland formation and activity *in vivo*. Analysis of meibomian gland morphology by light microscopy revealed marked differences between Bsg^−/−^ and WT mice in both the superior and inferior tarsal plates (Figure 4a). In WT mice, distinct clusters of secretory acini were observed throughout the length of the tarsal plates in the upper and lower lids. By comparison, secretory acini in meibomian glands of Bsg^−/−^ mice were poorly developed and appeared smaller in size (Figures 4b and c). Importantly, cells in secretory acini of Bsg^−/−^ mice failed to produce lipid vesicles as shown by Oil Red O staining (Figure 5a). In these experiments, the area of Oil Red O staining in tarsal plates of Bsg^−/−^ mice decreased by 76% and 84% in the superior and inferior lids, respectively (Figure 5b). These results indicate that CD147 expression is necessary and sufficient to induce meibocyte differentiation.

## Discussion

Despite the importance of the meibomian glands in promoting ocular surface health and integrity, research into molecular mechanisms regulating meibocyte formation and maturation is still limited. It is now well established that MMP biosynthesis is crucial for the development, differentiation, and activity of epithelial tissues.^8, 9, 24^ Here, we find that the metalloproteinase inducer CD147 is produced by secretory acini of human meibomian glands, in areas associated with high gelatinolytic activity. Notably, we identify CD147 as a novel regulator of MMP biosynthesis, cell proliferation, and cell differentiation in an experimental cell culture model with human meibocytes. In addition, we show that targeted deletion of CD147 in mice causes meibomian gland malformation and impairs meibocyte function *in vivo*.

MMPs play essential roles as mediators of change and physical adaptation in tissues, whether developmentally regulated, environmentally induced, or disease-associated.^9^ In line with previous reports in glandular tissues, recent data have demonstrated that regulation of MMP expression in ocular glands is important in the remodeling of the extracellular matrix during branching morphogenesis.^11, 25, 26^ Further, control of MMP activity is known to be critical to the maintenance of ocular surface health, and an improper balance of MMPs has been associated with inflammation and disease.^27^ To our knowledge, reports describing the distribution of gelatinolytic activity in the meibomian gland are absent. In the present study, we demonstrate by *in situ* zymography that MMP activity localizes to secretory acinar structures within the meibomian gland, both in proliferating and mature meibocytes, and decreases towards the disintegrating zone. These results showing gelatinolytic activity *in vivo* are consistent with previous data showing MMP expression in cultures of lipid-producing cells, including human meibocytes.^28, 29^ Importantly, they support a role for MMPs in regulating cellular homeostasis in the meibomian gland.

Numerous studies with experimental models have identified CD147 as one of the most important surface proteins responsible for the induction of MMPs.^12^ Ample evidence indicates that CD147 stimulates MMP synthesis through self-association, involving both heterotypic and homotypic cell–cell interactions.^13^ Although the mechanism by which CD147 regulates MMPs remains elusive, formation of CD147-based complexes, in which CD147 associates with additional proteins such as integrins, is known to modulate signaling and gene transcription, leading to distinct cellular responses.^30^ Our immunofluorescence staining of the human meibomian gland showed discrete clustering of CD147 along the cell membrane of differentiated meibocytes in glandular acini, which suggests self-association of CD147 in areas associated to gelatinolytic activity. Consistent with this hypothesis, we found that induction of meibocyte differentiation using serum-containing media in cell culture promoted CD147 clustering and stimulated MMP9 biosynthesis and secretion. Further, we demonstrated that supression of CD147 expression by siRNA reduced the levels of MMP9 in differentiated human meibocytes. Taken together, these results suggest that CD147 expression and clustering is involved in promoting gelatinolytic activity in differentiated meibocytes.

In addition to its role as an inducer of MMP biosynthesis, CD147 was recently found to be involved in the regulation of cell proliferation in tumor epithelial cells both *in vitro* and *in vivo.*^23, 31, 32^ In this investigation, we used BrdU incorporation and PCNA immunoblotting to assess the effects of CD147 expression on meibocyte proliferation *in vitro*. Consistent with previous reports, knockdown of CD147 reduced the proliferative activity of cells in culture. Moreover, differentiation was impaired as shown by decreased lipid accumulation in human meibocytes following CD147 abrogation. Histologic analysis of eyelids from Bsg^−/−^ mice also revealed that their meibomian glands were poorly developed and appeared smaller in size. In addition, the ability to produce lipids in the tarsal plated of Bsg^−/−^ mice was impaired compared with age-matched littermate controls. These findings strongly indicate that CD147 regulates the normal development and function of the meibomian gland. Lipids produced by the meibomian glands are a major component of the ocular surface, where they prevent tear evaporation and contribute to maintain a wet-surfaced phenotype.^19, 33^ Reduced quantity of lipids and increased protease activity on the ocular surface has been observed in patients with meibomian gland dysfunction.^4, 34^ Further, complete absence of meibomian glands has been reported in EEC syndrome and anhidrotic ectodermal dysplasia.^6^ It is tempting to speculate that CD147 has a role in the pathogenesis of patients suffering from these pathologies, but any hypothetical involvement remains to be determined.

To our knowledge, this is the first study to explore the role of CD147 in normal development and function of sebum-producing epithelial cells. Our findings indicate that CD147 plays a role in the induction and secretion of MMP9 during meibocyte differentiation. We find that decreased expression of CD147 reduces meibocyte cell proliferation and the ability to synthesize lipids. Finally, we demonstrate that the loss of CD147 results in impaired differentiation and malformation of the meibomian gland *in vivo*. We therefore propose a model where CD147 promotes meibomian gland turnover by regulating proteolytic activity and meibocyte maturation.

## Materials and Methods

### Cell culture and human tissue

Telomerase-transformed human meibomian gland epithelial cells were grown as previously described.^21^ Briefly, cells were grown in keratinocyte serum-free medium (Life Technologies, Carlsbad, CA, USA) to achieve confluence, followed by DMEM/F12 (Sigma-Aldrich, St Louis, MO, USA) supplemented with 10% calf serum for 7 days to promote differentiation. Experiments using human specimens were approved by the Schepens Eye Research Institute Institutional Review Board (IRB#2011-027). These studies were performed using discarded and deidentified human tissue and were deemed to have IRB exempt status not requiring informed consent. Discarded human eyelid tissue was collected from a donor who underwent ectropion repair and immediately frozen in optimal cutting temperature compound (OCT) for sectioning using a cryostat.

### Immunofluorescence

Sections of human eyelid tissue in OCT (6-*μ*m thick) were fixed with ice-cold methanol for 10 min and baked overnight at 37 °C. Following hydration in phosphate-buffered saline (PBS) for 10 min, sections were blocked in 3% bovine serum albumin in PBS for 30 min. After this, sections were incubated with primary anti-CD147 (clone HIM6,^35^ 1 : 50; BioLegend, San Diego, CA, USA), anti-PCNA (1 : 100; Santa Cruz Biotechnology, Inc., Santa Cruz, CA, USA), or anti-MMP9 (1 : 50; Abcam, Cambridge, MA, USA) antibodies in 3% BSA in PBS for 1 h at room temperature in a moist chamber. Incubation with primary antibodies was routinely omitted in control experiments. After rinsing with PBS, three times for 2 min each, the corresponding secondary antibodies were applied for 1 h at room temperature. Slides were then rinsed, mounted in VectaShield mounting medium containing DAPI (Vector Laboratories, Burlingame, CA, USA), and observed under a fluorescence microscope (Nikon Eclipse E-400; Tokyo, Japan).

### Laser capture microdissection

Sections of human eyelid tissue in OCT (6-*μ*m thick) were placed on Arcturus PEN Membrane Slides (Applied Biosystems, Foster City, CA, USA), fixed in 70% ethanol, and stained with Hematoxylin/Eosin (Sigma-Aldrich Co.) under RNase-free conditions. Meibomian gland secretory acini were captured using a laser microdissection microscope (Model AS LMD; Leica, Wetzlar, Germany) and subjected to total RNA extraction.

### Nucleic acid extraction and RT-PCR

Total RNA was isolated from laser-captured sections of meibomian gland acini using the RNeasy Micro Kit (Qiagen Sciences, Germantown, MD, USA), and 1 *μ*g of total RNA was used to make cDNA using the iScript cDNA synthesis kit (Bio-Rad Laboratories, Hercules, CA, USA) following the manufacturer's instructions. PCR reactions were performed with 0.8 *μ*l cDNA using the Advantage 2 PCR kit (Clontech Laboratories, Inc., Mountain View, CA, USA) and published CD147 primers.^36^ GAPDH primers (Applied Biosystems) were used as an internal control. Cultures of human corneal epithelial cells served as positive control. For CD147 amplification in laser-captured sections, the PCR reaction included an initial cycle of 95 °C for 5 min; 45 cycles of denaturation (95 °C for 30 s), annealing (64 °C for 30 s), and elongation (72 °C for 30 s); and a final extension cycle of 72 °C for 5 min. The PCR products (10 *μ*l) were analyzed electrophoretically in 1% (wt/vol) agarose gels containing ethidium bromide and visualized under UV light. To verify the identity of the CD147 PCR product, the band in the agarose gel was excised, and the extracted DNA was sequenced at the DNA Core Facility of Massachusetts General Hospital, Boston, MA, USA.

### Transfection of siRNA

Depletion of CD147 in cultures (6-well plates) of both proliferating and differentiated meibocytes was achieved using Silencer Select Pre-designed siRNA (ID215973; Ambion, Austin, TX, USA). Silencer Select Negative Control siRNA (ID4390843) was used as a scrambled sequence. For knockdown in proliferating cells, cultures grown to 60–70% confluence were transfected by 6-h incubation with 500 nM siRNA in Lipofectamine 2000 (1 *μ*l/100 mm^2^, Life Technologies) dissolved in Opti-MEM reduced-serum medium (Life Technologies). After transfection, the cells were incubated in serum-free medium lacking antibiotics for 18 h, followed by antibiotic-containing serum-free medium for an additional 48 h. For knockdown in differentiated cells and MMP analyses, cultures were grown to confluence and allowed to differentiate in serum-containing medium for 7 days. Thereafter, cells were transfected on days 7, 8, and 9 by 6-h incubation with 500 nM siRNA in Lipofectamine 2000 dissolved in serum-containing medium lacking antibiotics. After each transfection, cells were incubated in serum-containing medium lacking antibiotics for an additional 18 h. Cells in both proliferating and differentiated conditions were thoroughly washed in PBS and incubated in 1 ml serum-free medium for 3 h before collecting the supernatants and cell extracts. For knockdown in differentiated cells and LipidTOX analyses, cultures were transfected twice, once at 100% confluence, and then 3 days post confluence, and allowed to differentiate in serum-containing medium for 7 days. For each transfection, cells were treated with 500 nM siRNA in Opti-MEM reduced-serum medium in Lipofectamine 2000 for 6 h. Cultures were then incubated for 18 h with serum-containing medium lacking antibiotics. After transfections, the media was switched to serum-containing medium with antibiotics.

### Immunoblotting

Protein from cell cultures was extracted using RIPA buffer (150 *μ*M NaCl, 50 *μ*M Tris-HCl, pH 8.0, 1% NP-40, 0.5% deoxycholate, 0.1% SDS) supplemented with Complete Protease Inhibitor Cocktail (Roche Diagnostics, Indianapolis, IN, USA). After homogenization with a pellet pestle, the protein cell extracts were centrifuged at 12 000 × *g* for 45 min, and the protein concentration of the supernatant determined using the Pierce BCA Protein Assay Kit (Thermo Fisher Scientific, Rockford, IL, USA). Proteins in cell lysates (40 *μ*g) were resolved in 10% SDS-PAGE, and electroblotted onto nitrocellulose membranes (Bio-Rad). Membranes were then incubated with primary antibodies to CD147 (clone A-12,^37^ 1 : 3000, Santa Cruz Biotechnology, Inc.), MMP9 (1 : 1500, Abcam), PCNA (1 : 3000, Santa Cruz Biotechnology, Inc.) or GAPDH (1 : 3000, Santa Cruz Biotechnology, Inc.) in TTBS supplemented with 5% nonfat dry milk overnight at 4 °C, followed by the appropriate secondary antibodies coupled to horseradish peroxidase (1 : 5000, Santa Cruz Biotechnology, Inc.). Peroxidase activity was detected on HyBlot CL autoradiography film (Denville Scientific, Inc., Plainfield, NJ, USA) using SuperSignal West Pico Chemiluminiscence substrate (Thermo Scientific). Immunoblots were quantified using ImageJ software.

### *In situ* zymography

*In situ* gelatinase activity was measured using the EnzCheck Gelatinase Assay kit (Molecular Probes, Carlsbad, CA, USA) following the manufacturer's instructions. Briefly, sections of human eyelid tissue in OCT (6-*μ*m thick) were incubated with 2 *μ*g DQ-gelatin-fluorescein isothiocyanate overnight at 37 °C, washed with PBS and mounted using coverslips and VectaShield mounting medium containing DAPI (Vector Laboratories). Sections were then examined under a fluorescence microscope and photographed. As a negative control for *in situ* zymography, frozen cryostat sections were incubated with reaction buffer supplemented with 40 *μ*M 1,10-phenanthroline to inhibit metalloproteinases.

### Bromodeoxyuridine labeling

BrdU incorporation and flow cytometry analysis were used to assess cell cycle progression. Cultures of human meibocytes in serum-free media were stained using the APC BrdU Flow Kit (BD Pharmingen, San Jose, CA, USA) according to the manufacturer's protocol. Briefly, cells were harvested 12 h following addition of 10 *μ*M BrdU into the culture media. Following fixation and permeabilization, incorporated BrdU was exposed by treatment with DNase for 1 h at 37 °C. Cells were subsequently stained with APC-anti-BrdU antibody (1 : 50) and analyzed on a BD LSRII flow cytometer (Beckton Dickenson, San Jose, CA, USA). Cells from the same population that were not exposed to BrdU were used as a negative staining control to determine background-staining levels for the anti-BrdU monoclonal antibody.

### Mouse tissue

Animals used in this research were maintained in accordance with the guidelines of the Institutional Animal Care and Use Committee at the University of Illinois. The WT C57BL6/J and basigin-null (Bsg^−/−^) mice^38, 39^ were housed under temperature- and light-controlled conditions (12 h light: 12 h dark) with free access to food and water. Adult mice (10–14-months old) were anesthetized by isoflurane inhalation (Attane, Minrad, Bethlehem, PA, USA). For histological studies, mice heads were fixed in 1% glutaraldehyde in PBS. Eyes with intact lids were then excised and embedded in methacrylate. Semithin sections (3-*μ*m thick) were stained with toluidine blue for light microscopy. For lipid staining and fluorescence microscopy, mice heads were fixed in 2% PBS-buffered formalin and the excised eyes with intact lids embedded in OCT. All mouse tissue was sectioned at the central eyelid position. Photomicrographs were taken using Nikon Eclipse E-800 microscope (Nikon Instruments, Melville, NY, USA).

### LipidTOX green and oil red o staining

For lipid staining in cell culture, cells grown on glass chamber slides (Nalgene Nunc International, Naperville, IL, USA) were washed with PBS and fixed in 4% paraformaldehyde for 30 min. Slides were incubated in LipidTOX Green neutral lipid stain (Invitrogen, Grand Island, NY, USA) in a moist chamber for 30 min at room temperature. Slides were then mounted in VectaShield mounting medium containing DAPI (Vector Laboratories), and observed under a fluorescence microscope. Quantification of lipid staining was performed using imageJ software (NIH, Bethesda, MD, USA). For lipid staining in mouse tissue, cryosections (10-*μ*m thick) were air-dried, rinsed in 60% isopropanol, and stained with 0.3% Oil Red O (Fisher Biotec, Wembley, WA, Australia) for 15 min at room temperature. The sections were rinsed again and mounted in VectaShield mounting medium for light microscopy.

## Abbreviations

BrdU: bromodeoxyuridine
Bsg: basigin
CD147: cluster of differentiation 147
DAPI: 4',6-diamidino-2-phenylindole
DMEM/F12: Dulbecco's modified Eagle medium: nutrient mixture F-12
ECC: ectrodactyly ectodermal dysplasia and cleft lip/palate
GAPDH: glyceraldehyde 3-phosphate dehydrogenase
hTERT: human telomerase reverse transcriptase
LCM: laser capture microdissection
MMPs: matrix metalloproteinases
NP-40: Nonidet P-40
OCT: optimal cutting temperature compound
PAGE: polyacrylamide gel electrophoresis
PBS: phosphate-buffered saline
PCNA: proliferating cell nuclear antigen
RNAi: RNA interference
RIPA: radioimmunoprecipitation assay
RT-PCR: reverse transcription-polymerase chain reaction
SD: standard deviation
SDS: sodium dodecyl sulfate
siRNA: small interfering RNA
UV: ultraviolet
WT: wild type
